# Symptoms and levels of ICD-11 Prolonged Grief Disorder in a representative community sample of UK adults

**DOI:** 10.1007/s00127-023-02469-1

**Published:** 2023-04-11

**Authors:** Mark Shevlin, Enya Redican, Philip Hyland, Jamie Murphy, Thanos Karatzias, Orla McBride, Kate Bennett, Sarah Butter, Todd K. Hartman, Frédérique Vallières, Richard P. Bentall

**Affiliations:** 1 School of Psychology, Ulster University, Cromore Road, Coleraine, BT52 1SA Northern Ireland; 2 Maynooth University, Maynooth, Ireland; 3 Napier University, Edinburgh, Scotland; 4 University of Liverpool, Liverpool, England; 5 University of Manchester, Manchester, England; 6 Centre for Global Health, Trinity College Dublin, Dublin, Ireland; 7 University of Sheffield, Sheffield, England

**Keywords:** Prolonged Grief Disorder, Epidemiology, Prevalence, Risk factors

## Abstract

**Background:**

Prolonged Grief Disorder (PGD) is a new disorder included in ICD-11 (WHO, 2018). There is a growing body of literature surrounding the prevalence and correlates of ICD-11 PGD symptoms as assessed using various measures. This study was the first to assess levels of ICD-11 PGD symptoms as measured by the International Prolonged Grief Disorder Scale (IPGDS), a self-report scale directly aligned with the ICD-11 definition of PGD, among the United Kingdom adult general population, and identify correlates.

**Method:**

Participants included 2025 adults who participated in Wave 5 of the COVID-19 Psychological Research Consortium Study (C19PRC-UK). Prevalence rates of PGD were estimated based on two commonly used algorithms defined as ‘strict’ and ‘moderate’. Sociodemographic, loss-related, and mental health correlates (i.e., anxiety, depression, mental health treatment seeking, loneliness) of strict and moderate PGD were then examined using multinomial logistic regressions.

**Results:**

It was found that 2.4% (n = 43) of participants met probable caseness for PGD using the strict criteria while 7.9% (n = 140) met probable caseness for PGD using the moderate criteria. Multinomial logistic regression analysis results showed, as predicted, that income, time since bereavement, death of a child, religiosity, and depression were associated with both moderate and strict PGD. Correlates of moderate PGD included country of residence, urbanicity, younger age of bereaved, and loneliness.

**Conclusions:**

This study highlights that some symptoms of PGD are commonly reported in the general population, although relatively few meet the criteria for clinical significance. The routine assessment for PGD following a bereavement is discussed and the development of appropriate interventions are recommended.

## Introduction

The loss of a loved one is a common and often highly stressful human experience. Although most people ultimately adapt to their loss [1], there is a sizeable minority of bereaved individuals who experience an enduring and disabling grief response [2]. This occurrence of maladaptive grief responses has been recognized in the most recent version of the International Classification of Diseases [ICD-11; 3] via the introduction of Prolonged Grief Disorder (PGD). Essential characteristics of PGD include (i) a history of bereavement, (ii) persistent and pervasive longing for, or preoccupation with, the deceased associated with intense emotional pain (e.g., sadness, guilt, denial), (iii) functional impairment, and (iv) symptoms persisting for an atypically long time relative to cultural norms [3].

Recently, the International Prolonged Grief Disorder Scale (IPGDS; 4) was developed as the first self-report measure of ICD-11 PGD symptoms. The IPGDS is a pan-cultural measure designed for the assessment of the ICD-11 PGD diagnosis. Despite being a novel instrument, emerging research provides support for the psychometric properties of the scale [4, 5]. This scale can generate scores to reflect PGD severity, but there is a lack of consensus regarding how best to identify levels of PGD that may require clinical help. Two approaches to identifying probable caseness have been proposed for the IPGDS, the ‘moderate’ and ‘strict’ approaches (hereafter referred to as ‘moderate PGD’ and ‘strict PGD’ respectively) [4, 5]. The required frequency of symptoms varies between the two approaches; for moderate PGD, symptoms must appear “sometimes”, “often”, or “always”, and for strict PGD, symptoms must appear “frequently”, or “always”. There has been limited research on the levels of ‘moderate PGD’ and ‘strict PGD’ as assessed using the IPGDS in samples of bereaved adults, with prevalence estimates ranging from 19.8 to 33.5% for moderate, and 6.9 to 37.8% for strict (in adult samples from Germany, China, Switzerland, and Portugal [4, 5]. To date, no study has examined the prevalence or correlates of moderate and strict PGD as measured by the IPGDS in a representative general population sample from the United Kingdom (UK).

It should be emphasized that various instruments have been developed for the assessment of ICD-11 PGD symptoms, including the extended version of the Prolonged Grief Disorder-13 (PG13 + 9; 6) and the Traumatic Grief Inventory-Self Report Plus (TGI-SR+; 7). The “strict” approach—also referred to as ‘liberal’ approach—is used by both the PG13 + 9 and the TGI-SR + , while the TGI-SR + also offers the option of a ‘conservative’ approach, which increases the number of symptoms necessary for diagnosis. Other studies have used proxy items derived from established measures to measure ‘strict’ and ‘conservative’ PGD [e.g., 8, 9]. Prevalence rates of probable ‘strict PGD’ have ranged from 1.5% in the German general population to 34% in a community sample of bereaved Danish adults [6, 7, 9]. Establishing prevalence rates of both probable ‘strict’ and ‘moderate’ PGD using different measures including the IPGDS is necessary to determine the diagnostic concordance between these measures. This is especially important considering how much emphasis the grief literature has placed on determining the best method for diagnosing PGD. There are currently five different criteria sets proposed within the literature, each of which generates diverging PGD prevalence rates [10]. Hence, more research is needed to determine the best way to implement the ICD formulation of PGD to ensure consistency in prevalence estimates across studies. Finally, it is important to consider the potential influence of the COVID-19 pandemic on prevalence rates. Emerging research indicates elevated levels of PGD among individuals bereaved during the COVID-19 pandemic [e.g., 11, 12], likely attributable to more unexpected deaths, changes to customary mourning and grief rituals, and lack of social support due to government restrictions [13].

This study aimed to report the levels of PGD symptoms, and estimate rates of moderate/strict PGD, in the UK general population and identify significant correlates. It was anticipated that correlates of PGD would be similar to those identified in other countries including sociodemographic factors such as gender [14], unemployment, low income, presence of children in the household [15], and being in a committed relationship [17]. It was anticipated that less time since passing [9, 14] and younger age of the deceased [16, 17] would be significant correlates of PGD. Finally, it was anticipated that mental-health factors would be significantly associated with PGD including depression [15–17], and loneliness [e.g., 18]. The present study also sought to look at the association between prior mental health treatment and PGD, however, no a priori hypotheses were developed due to the paucity of research in this area.

## Methods

### Participants

This study used data from wave five of the COVID-19 Psychological Research Consortium Study (C19PRC-UK), a longitudinal internet-based survey assessing the psychological and socioeconomic impact of the COVID-19 pandemic [19]. The C19PRC data have been discussed extensively elsewhere [20]. In brief, data for wave one of the C19PRC study was collected between the 23rd and 28th of March 2020 through the survey company Qualtrics. Quota sampling methods, a non-probability based sampling method whereby quotas of participants with certain characteristics are obtained, were used to ensure national representativity of the sample regarding age, sex, and household income. The final survey sample comprised of 2025 adults aged 18 years and above. Data collection for wave five of the C19PRC-UK study began on the 22nd of March 2021, approximately one year following the completion of the baseline survey [20]. Data collection was conducted in two phases: in phase 1 (24th March to 20th April 2021) adults who participated in Wave 4 of the survey (n = 3867) were contacted by Qualtrics either by email, SMS, or by in-app notifications and invited to participate further in the survey. In phase 2 (8th April to 20th April 2021) participants who had completed any other wave (i.e., waves one to three) were recontacted and invited to participate in wave five. Phase one of this fieldwork resulted in a total of 2377 participants from wave four completing wave five (61.5% recontact rate), while phase 2 resulted in an additional 143 participants from wave one to wave three re-entering the survey [19]. In total, there were 2520 participants for Wave 5. For the purposes of the present study, only those who were bereaved were eligible for inclusion. Specifically, respondents were asked “*At any time in your life, has someone close to you died (e.g., a partner, parent, child, friend)?”* and “*When did the death occur*?”. If the respondent indicated ‘yes’ to the former and more than six months ago to the latter, they were screened in to complete the IPGDS. Hence, the current study has a final sample size of 2025 participants. Ethical approval for the study was granted by the University of Sheffield (Ethical approval ref no. 033759). The wave five data used in the current study is available at: https://osf.io/ducgs.

### Measures

#### ICD-11 Prolonged Grief Disorder

The International Prolonged Grief Disorder Scale (IPGDS; 4) is a fourteen-item self-report measure of ICD-11 PGD symptoms. The IPGDS contains two items measuring core symptoms (i.e., “*longing or yearning”* and *“preoccupied with thoughts*”) and an additional ten items measuring associated emotional symptoms including anger, avoidance, sorrow, and emotional numbness (see Table 1; items 3–12). One item measures functional impairment, while another measures the extent to which symptoms deviate from community or cultural norms. Participants rate symptom frequency during the past week using a five-point Likert scale ranging from ‘Not at all’ (1) to ‘Always’ (5). The reliability of the twelve symptom items of the IPGDS in the current study was *α* = 0.94.

**Table 1:** Endorsement rates for IPGDS items

	Moderate			Strict		
	N	%	95% CI	N	%	95% CI
Item 1: I am longing or yearning for the deceased	832	46.8	(44.6, 49.2)	230	12.9	(11.4, 14.5)
Item 2: I am preoccupied with thoughts about the deceased or circumstances of the death	522	29.4	(27.1, 31.6)	150	8.4	(7.1, 9.7)
Item 3: I have intense feelings of sorrow, related to the deceased	749	42.1	(39.9, 44.3)	276	15.5	(13.9, 17.1)
Item 4: I feel guilty about the death or circumstances surrounding the death	401	22.6	(20.6, 24.5)	136	7.7	(6.5, 8.8)
Item 5: I am angry over the loss	498	28.0	(25.9, 30.1)	191	10.7	(9.3, 12.2)
Item 6: I try to avoid reminders of the deceased or the death as much as possible (e.g., pictures, memories)	396	22.3	(20.3, 24.4)	126	7.1	(6.0, 8.3)
Item 7: I blame others or the circumstances for the death (e.g., a higher power)	249	14.0	(12.3, 15.7)	83	4.7	(3.7, 5.6)
Item 8: I have trouble or just don’t want to accept the loss	405	22.8	(20.8, 24.8)	141	7.9	(6.8, 9.1)
Item 9: I feel that I lost a part of myself	716	40.3	(38.0, 42.7)	294	16.5	(14.8, 18.3)
Item 10: I have trouble or have no desire to experience joy or satisfaction	348	19.6	(17.8, 21.4)	126	7.1	(6.0, 8.2)
Item 11: I feel emotionally numb	414	23.3	(21.2, 25.4)	119	6.7	(5.6, 7.8)
Item 12: I have difficulties engaging in activities I enjoyed prior to the death	297	16.7	(14.8, 18.5)	88	5.0	(4.0, 5.9)
Additional Items						
Item 13: Grief significantly interferes with my ability to work, socialize or function in everyday life	247	13.9	(12.2, 15.7)	80	4.5	(3.7, 5.5)
Item 14: My grief would be considered worse (e.g., more intense, severe and/or of longer duration) than for others from my community or culture	260	14.6	(12.9, 16.4)	97	5.5	(4.5, 6.5)

### Correlates

#### Bereavement timeframe

The IPGDS includes an additional item which asks participants to indicate when their loss occurred. The following response options are provided: (a) less than 6 months ago, (b) 6–12 months ago, (c) 1–5 years ago, (d) 5–10 years ago, (e) 10–20 years ago, and (f) more than 20 years ago. Because the current study included only those bereaved longer than six months (due to the requirement for symptoms to be present for ≥ 6 months in the ICD-11) the first category was redundant. The final two categories were collapsed into a single category because of low numbers of participants in each, leaving a total of four categories for the time since bereavement variable (1 = 6–12 months, 2 = 1–5 years, 3 = 5–10 years, 4 =  > 10 years).

#### Age of deceased

Participants were also asked “*how old was the person when they died*?”, with responses reported in years. A multi-categorical variable was created to represent the age group of the deceased (i.e., 1 = 0–17 years, 2 = 18–34 years, 3 = 35–59 years, 4 = 60–79 years, 5 = 80 years and above).

#### Demographics

Participants indicated their sex (0 = male, 1 = female) and age which was collapsed into four categories (18–34 years; 35–44 years; 45–54 years; 65 years and over).

#### Living location

Area (city, suburb, town, rural) and country (England, Scotland, Wales, Northern Ireland) of residence were assessed.

#### Relationship status

Participants were asked to indicate their current relationship status using six categories (single - not currently in a committed relationship; in a committed relationship but not living together; cohabiting; married; in a civil partnership; single- never been in a committed relationship). Relationship status was recoded into a binary variable for the purposes of the current study (0 = not in a committed relationship, 1 = in a committed relationship).

#### Religious identify

Participants were asked to indicate their religious identity and were provided with eleven response options: Atheist, Agnostic, Catholic, Protestant, Sunni, Shia, Jewish, Buddhist, Sikh, Hindu, Other. The data was recoded into a binary variable representing religious status [0 = not religious (i.e., Atheist, Agnostic), 1 = religious (i.e., all other categories)].

#### Parental status

Participants were asked to identify their parental status; categories included: (1) I do not have any children; (2) I have a child/children under 18 years of age, and he/she/they primarily live with me in my household; (3) I have a child/children under 18 years of age, but he/she/they primarily live elsewhere; (4) I have a child/children aged 18 years or over, and he/she/they primarily live with me in my household; (5) I have a child/children aged 18 years or over, but he/she/they primarily live elsewhere, and (6) someone else’s child/children under 18 years of age live with me in my household. The data were recoded into a binary variable to represent parental status (0 = child/children 18 years or below in the household (i.e., 1, 3, 4, 5), 1 = child/children 18 years or above in the household (i.e., items 2, 6).

#### Finances and employment

Participants were asked to indicate their self-estimated gross annual income for 2019 (1 = £0–15,490; 2 = £15, 491–£25, 340; 3 = £25,341–£38,740; 4 = £38,741–£57,930; 5 = £57,931 or more) and if they were currently in receipt of any government benefits excluding child support and state pension (0 = not in receipt, 1 = in receipt). Current employment status was initially measured using 10 categories and was recoded into a binary variable representing economic activity (0 = not economically active (unemployed, retired, full-time student), 1 = economically active (employed full or part-time, self-employed full or part-time, furloughed due to COVID-19 Pandemic).

### Mental health

#### Depression

Symptoms of major depressive disorder (MDD) were assessed using the PHQ-9 [21]. Participants were asked to indicate how often, over the last two weeks, they had been bothered by each of the nine symptoms contained within the measure. Responses ranged from ‘not at all’ (0) to ‘nearly every day’ (3). Possible PHQ-9 scores ranged from 0 to 27, with higher scores indicative of higher levels of depression symptomology. Scores of ≥ 5, ≥ 10, and ≥ 15 indicate mild, moderate, and severe levels of depression, respectively (13), with the recommended cut-off score of ≥ 10 used in the current study to indicate meeting caseness criteria [22]. This cut-off score has been shown to have adequate sensitivity (0.85) and specificity (0.89) for detecting cases of MDD [23]. The psychometric properties of the PHQ-9 scores have been widely supported [23] and the internal consistency of the scale in this study was *α* = 0.92.

#### Anxiety

The GAD-7 [23] was used to measure symptoms of generalized anxiety disorder (GAD). Participants were asked to indicate, using a 4-point Likert scale ranging from ‘not at all’ (0) to ‘nearly every day’ (3), the extent to which they were bothered by each of the seven anxiety symptoms contained within the GAD-7 over the past week. Possible GAD-7 scores range from 0 to 21, with higher scores indicating higher levels of anxiety symptomology. For the current study, the recommended cut-off score of ≥ 10 was used to indicate clinically significant anxiety symptoms as this cut-off score has been shown to have adequate sensitivity (0.89) and specificity (0.82) for detecting cases of GAD [24]. The validity and the reliability of the GAD-7 as a measure of anxiety has been supported for use in the general population [24]. The internal consistency of the GAD-7 in the present study was *α* = 0.95.

#### Mental health treatment seeking

Participants were presented with the following statement: “*mental health difficulties are very common. It will help us understand our survey results if you would tell us whether you currently or have in the past received treatment (medication or talking therapies) for these kinds of difficulties.*” Participants were required to tick all options that applied to them which included: (1) I have never received treatment for mental health problems, (2) I have received treatment for mental health problems in the past, (3) I am currently receiving treatment for mental health problems, (4) I am currently receiving treatment for mental health problems but it has been cancelled temporarily due to the lockdown, (5) I am currently on a waiting list to receive treatment for a mental health problem, and (6) Prefer not to answer”. A binary variable was created to represent current mental health help seeking [0 = no (1, 2, 6), 1 = yes (3, 4, 5)].

#### Loneliness

Loneliness was measured using the Loneliness Scale [25], a three-item measure specifically designed for use in large-scaled population surveys. Respondents were asked how often they felt (no time frame specified): (1) that they lacked companionship, (2) left out; and (3) isolated from others. Responses were scored on a three-point Likert scale ranging from ‘hardly ever’ (1) to ‘often’ (3). Possible scores ranged from 3 to 9, with higher scores indicating higher levels of loneliness. A cut-off score of 6 or more was used to indicate loneliness in the present study, as has been done in prior research [e.g., 26]. The internal consistency of the Loneliness Scale in the current study was *α* = 0.90.

### Analytic procedure

#### Diagnostic algorithms

Although the ICD-11 lists essential requirements for PGD it does not specify the number or severity of symptoms required to meet the clinical criteria. Two diagnostic algorithms have been proposed to date [4, 5]. PGD requires the (1) endorsement of one or more core symptom, (2) one or more emotional symptom, (3) significant functional impairment, and (4) the grief response persisting beyond cultural or community norms. For the IPGDS the ‘strict’ requirement is met when the items are rated ≥ 4 (i.e., ‘often’, ‘always’) on the Likert scale, and ≥ 3 (i.e., ‘sometimes’, ‘often’, ‘always’) for ‘moderate’.

### Statistical analysis

The frequencies of PGD symptom-endorsement and the proportions of participants meeting requirements for ICD-11 PGD according to the ‘strict’ and ‘moderate’ algorithms were calculated. Next, a tripartite variable was created representing whether a participant met probable caseness using the (1) strict or (2) moderate criterion, or (3) did not meet probable caseness for either. Chi-square tests of association were conducted to examine the association between each correlate and this grouping variable, with the strength of these associations quantified using Cramer’s *V* (≤ 0.2 = weak, 0.2–0.6 = moderate, > 0.6 strong). Adjusted standardised residuals ≥ 1.96 for cells in the cross-tabulations were used to assess significant differences between the observed and expected counts. Following this, all multi-categorical correlates were dummy coded. The bivariate associations between each of the correlates variables and the three-level dependent variable were calculated using multinomial logistic regressions, with the ‘no criteria’ group serving as the reference category. Results are reported as odds ratios (ORs) with 95% confidence intervals. Finally, all correlates were then entered simultaneously in the logistic regression to examine the effects of each correlate while controlling for all other potentially influential correlates, with results reported as adjusted ORs (AOR) with 95% confidence intervals; this was the multivariate model.

## Results

### Symptoms and levels of PGD

Of the 2,025 participants, 1771 (70.5%) had reported having lost someone more than 6 months ago. The frequencies of PGD symptom-endorsement are presented in Table 1.The most commonly endorsed PGD symptoms at both the moderate and strict level were “*longing or yearning for the deceased*” (moderate = 46.8%; strict = 12.9%), “*intense feelings of sorrow, related to the deceased*” (moderate = 42.1%; strict = 15.5%), “*lost a part of myself*” (moderate = 40.3%; strict = 16.5%), and “*preoccupied with thoughts about the deceased or circumstances of the death*” (moderate = 29.4%; strict = 8.4%). The least commonly endorsed PGD symptoms at both the moderate and strict level were “*grief significantly interferes with my ability to work, socialize or function in everyday life*” (moderate = 13.9%; strict = 4.5%), “*my grief would be considered worse than for others from my community or culture*” (moderate = 14.6%, strict = 5.5%) *and* “*I blame others or the circumstances for the death*” (moderate = 14.0%, strict = 4.7%)*.* In this sample, 7.9% (95% CI 6.6, 9.1: n = 140) met probable caseness using the moderate criteria, and 2.4% (95% CI 1.7, 3.1: n = 43) met probable caseness using the ‘strict’ criteria for ICD-11 PGD.

### Chi square tests of association results

Chi-square tests of association were conducted to examine the bivariate relationship between the correlates and diagnostic status for those participants who were bereaved for longer than six months (see Table 2). All correlates were significantly associated with diagnostic status except for gender, religious status, country, and area of residence. For the sociodemographic variables the largest effect sizes were for being in receipt of benefits (*V* = 0.14) and Age (*V* = 0.13: significantly more 65 + years in the moderate and strict criteria groups), and these effects were small. The effects sizes for the psychosocial variables were larger with participants screening positive for anxiety (*V* = 0.27), depression (*V* = 0.32), loneliness (*V* = 0.23), and mental health help-seeking (*V* = 0.16) all more likely to be in the moderate or strict PGD groups. The effect sizes were generally moderate.

**Table 2:** Crosstabulation of diagnostic status and sociodemographic, loss-related and mental health factors

	N (%)	χ^2^ (df)	ES	No PGD (n = 1594)		Moderate criteria (n = 140)		Strict criteria (n = 43)	
				N	(%)	N	(%)	N	(%)
Income		8.44 (2)*	0.09						
£0–£15,490 per annum	358 (20.1%)			***308***	***(19.3)***	34	(24.3)	***16***	***(37.2)***
£15,491–£25, 340 per annum	333 (18.7%)			***285***	***(17.9)***	***39***	***(27.9)***	9	(20.9)
£25,341–£38,740 per annum	395 (22.2%)			354	(22.2)	34	(24.3)	7	(16.3)
£ 38.741–£57,930 per annum	363 (20.4%)			***336***	***(21.1)***	20	(14.3)	7	(16.3)
£57,931 or more per annum	328 (18.5%)			***311***	***(19.5)***	***13***	***(9.3)***	4	(9.3)
Benefits		33.12 (2)**	0.14						
Yes	358 (20.1%)			***295***	***(18.5)***	***42***	***(30.0)***	***21***	(48.8)
Employment status		8.44 (2)**	0.07						
Economically active	917 (51.6%)			***810***	***(50.8)***	***88***	***(62.9)***	19	(44.2)
Country		8.70 (6)	n/a						
England	928 (52.2%)			844	(52.9	62	(44.3)	22	(51.2)
Wales	303 (17.1%)			272	(17.1)	23	(16.4)	8	(18.6)
Scotland	334 (18.8%)			298	(18.7)	28	(20.0)	8	(18.6)
Northern Ireland	212 (11.9%)			***180***	***(11.3)***	***27***	***(19.3)***	5	(11.6)
Area of residence		12.10 (6)	n/a						
City	305 (17.2%)			***263***	***(16.5***	***33***	***(23.6)***	9	(20.9)
Town	533 (30.0%)			469	(29.4)	50	(35.7)	14	(32.6)
Suburb	541 (30.4%)			491	(30.8)	37	(26.4)	13	(30.2)
Rural area	398 (22.4%)			***371***	***(23.3***)	***20***	***(14.3)***	7	(16.3)
Age of participant		60.11 (8)**	0.13						
18–34	206 (11.6%)			***163***	***(10.2***)	***34***	***(24.3)***	9	(20.9)
35–44	247 (13.9%)			***210***	***(13.2***)	***31***	***(22.1)***	6	(14.0)
45–54	349 (19.6%)			309	(19.4)	28	(20.0)	12	(27.9)
55–64	483 (27.2%)			438	(27.5)	31	(22.1)	14	(32.6)
65+	492 (27.7%)			***474***	***(29.7***)	***16***	***(11.4)***	***2***	***(4.7)***
Gender		1.68 (2)	n/a						
Female	883 (49.9%)			788	(49.5)	70	(50.7)	59.5	(20.9)
Relationship status		60.11 (8)**	0.08						
In a committed relationship	1232 (69.3%)			***1123***	***(70.5***)	***86***	***(61.4)***	***23***	***(53.5)***
Parental status		10.14 (2)**	0.08						
Child/children under 18 years living in the household	317 (17.8%)			***269***	***(16.9***)	***38***	***(27.1)***	33	(23.4)
Other	1460 (82.2%)			***1325***	***(83.1***)	***102***	***(72.9)***	10	(76.7)
Religious		1.84 (0.2)	0.03						
Religious	1125 (63.3%)			1001	(62.8)	94	(67.1)	30	(69.8)
Time since bereavement		43.36 (8)**	0.11						
6–12 months	150 (8.4%)			***124***	***(7.8)***	***20***	***(14.3)***	6	(14.0)
1–2 years	214 (12.0%)			***173***	***(10.6)***	***27***	***(19.3)***	***14***	***(32.6)***
2–5 years	390 (21.9%)			356	(22.3)	27	(19.3)	7	(16.3)
5–10 years	381 (21.4%)			341	(21.4)	30	(21.4)	10	(23.3)
10 + years	642 (36.1%)			***600***	***(37.6)***	***36***	***(25.7)***	***6***	(14.0)
Age of deceased		37.68 (8)**	0.10						
0–17 years	44 (2.5%)			***34***	***(2.1)***	6	(4.3)	***4***	***(9.3)***
18–34 years	66 (3.7%)			***52***	***(3.3)***	***10***	***(7.2)***	***4***	***(9.3)***
35–59 years	308 (17.4%)			***264***	***(16.6)***	30	(21.6)	***14***	***(32.6)***
60–79 years	680 (38.4%)			612	(38.5)	54	(38.9)	14	(32.6)
80 years or above	675 (38.1%)			***629***	***(39.5)***	***39***	***(28.1)***	***7***	***(16.3)***
Anxiety		132.46 (8)**	0.27						
Meets caseness criteria	284 (16.0%)			***205***	***(12.9)***	***51***	***(36.4)***	***28***	***(65.1)***
Depression		184.313 (2)**	0.32						
Meets clinical caseness	362 (20.4%)			***258***	***(16.2)***	***70***	***(50.0)***	***34***	***(79.1)***
Loneliness		91.786(2)**	0.23						
Yes	642 (36.1%)			***517***	***(32.4)***	***97***	***(69.3)***	***28***	***(65.1)***
Mental health help-seeking		46.09(2)**	0.16						
Yes	449 (25.3%)			***365***	***(22.9)***	***65***	***(46.4)***	***19***	***(44.2)***

ES = Cramer’s *V* (≤ 0.2 = weak, 0.2–0.6 = moderate, > 0.6 strong), standardised residuals > 1.96 in bold

### Regression results

The bivariate association between each of the correlates and group membership were examined (see Table 3). The reference group for all analyses was ‘no criteria met’ group. When the unadjusted ORs were calculated, significant positive correlates of moderate PGD included lower income, receiving government benefits, being economically active, living in a city or town, living in Northern Ireland, younger age, having a child/children under 18 years living in the household, younger age of deceased, and all mental health variables. Regarding strict PGD membership, significant positive correlates in the unadjusted analyses included earning between £0–£15,490 per annum, receiving government benefits, younger age, younger age of deceased, and all mental health variables. Significant negative correlates of strict PGD included being in a committed relationship.

**Table 3:** Correlates of ICD-11 PGD diagnostic status (unadjusted and adjusted) for bereaved for greater than six months sample

	Moderate criteria				Strict criteria			
	OR	CI	AOR	AOR CI	OR	CI	AOR	AOR CI
Income								
£0–15,490 per annum	2.64**	(1.37, 5.10)	2.24*	(1.00, 5.00)	4.04*	(1.34, 12.22)	1.41	(0.35, 5.68)
£15,491–£25,340 per annum	3.27**	(1.71, 6.26)	2.25*	(1.08, 4.69)	2.46	(0.75, 8.06)	1.06	(0.26, 4.27)
£25,341–£38,740 per annum	2.30*	(1.19, 4.43)	1.92	(0.92, 3.99)	1.54	(0.45, 5.30)	1.55	(0.39, 6.21)
£38,741–£57,930 per annum	1.42	(0.70, 2.91)	1.13	(0.52, 2.45)	1.62	(0.47, 5.59)	1.16	(0.28, 4.70)
£57,931 or more per annum	–	–	–	–	–			
Benefits								
Yes	1.89**	(1.29, 2.77)	1.18	(0.72, 1.95)	4.20**	(2.28, 7.75)	2.17	(0.95, 4.97)
Economic activity								
Employed (economically active)	1.64**	(1.15, 2.34)	1.48	(0.91, 2.41)	0.77	(0.42, 1.41)	0.65	(0.28, 1.52)
Country								
Scotland (yes)	1.15	(0.70, 1.89)	1.72	(0.91, 2.57)	1.13	(0.50, 2.56)	1.49	(0.57, 3.91)
Wales (yes)	1.28	(0.80, 2.04)	1.53	(0.91, 2.57)	1.03	(0.45, 2.34)	1.23	(0.47, 3.16)
Northern Ireland (yes)	2.04**	(1.26, 3.30)	2.22**	(1.27, 3.88)	1.07	(0.40, 2.85)	0.86	(0.24, 3.12)
England	–	–	–	–	–	–	–	–
Area of residence								
City	2.33**	(1.31, 4.15)	1.51	(0.79, 2.89)	1.81	(0.67,4.93)	1.39	(0.40, 4.87)
Town	1.98**	(1.16, 3.38)	1.85*	(1.03, 3.34)	1.58	(0.63, 3.96)	2.07	(0.67, 6.43)
Suburb	1.40	(0.80, 2.45)	1.14	(0.62, 2.09)	1.40	(0.55, 3.55)	1.33	(0.43, 4.12)
Rural	–	–	–	–	–	–	–	–
Age category	–	–	–	–	–	–	–	–
18–34	6.18**	(3.32, 11.49)	2.40*	(1.06, 5.43)	13.09**	(2.80, 61.19)	3.37	(0.55, 20.80)
35–44	4.37**	(2.34, 8.17)	1.93	(0.87, 4.25)	6.77*	(1.36, 33.83)	2.26	(0.35, 14.66)
45–54	2.68**	(1.43, 5.04)	1.23	(0.57, 2.63)	9.20**	(2.05, 41.41)	3.23	(0.59, 17.60)
55–64	2.10**	(1.13, 3.89)	1.38	(0.69, 2.76)	7.58**	(1.71, 33.52)	4.68	(0.93, 23.58)
65 +	–	–	–	–	–	–	–	–
Gender								
Female	1.05	(0.74, 1.49)	0.61	(0.41, 0.90)	1.50	(0.80, 2.80)	1.06	(0.51, 2.21)
Relationship	–	–	–	–	–	–	–	–
In a committed relationship	0.67*	(0.47, 0.95)	1.08	(0.69, 1.67)	0.48*	(0.26, 0.89)	0.73	(0.33, 1.62)
Religious								
Yes	1.21	(0.839, 1.75)	1.63*	(1.08, 2.45)	1.37	(0.71, 2.64)	2.15*	(1.01, 4.59)
Household composition								
Children under 18 years in the household	1.84**	(1.24, 2.72)	1.55	(0.95, 2.55)	1.49	(0.73, 3.07)	1.19	(0.48, 2.96)
Time since bereavement								
6–12 months	**2.69**	(1.51, 4.80)	2.04*	(1.07, 3.89)	4.84**	(1.54, 15.25)	4.91*	(1.34, 18.08)
1–2 years	**2.60**	(1.54, 4.41)	1.94*	(1.07, 3.52)	8.09**	(3.06, 21.37)	9.43**	(3.04, 29.25)
2–5 years	1.26	(0.76, 2.12)	1.04	(0.59, 1.86)	1.97	(0.66, 5.90)	2.86	(0.84, 9.76)
5–10 years	1.46	(0.89, 2.42)	1.24	(0.71, 2.17)	2.93*	(1.06, 8.14)	2.79	(0.35, 5.68)
10 + years	–	–	–	–	–	–	–	–
Age of deceased								
0–17 years	2.85*	(1.13, 7.19)	2.94*	(1.05, 8.25)	10.57**	(2.95, 37.87)	14.22**	(2.76, 73.42)
18–34 years	3.10**	(1.47, 6.57)	2.20	(0.95, 5.09)	6.91**	(1.96, 24.38)	6.55*	(1.53, 28.00)
35–59 years	1.83*	(1.12, 3.01)	1.44	(0.82, 2.53)	4.77**	(1.90, 11.94)	3.83*	(1.36, 10.80)
60–79 years	1.42	(0.93, 2.18)	1.31	(0.82, 2.08)	2.06	(0.82, 5.13)	1.69	(0.63, 4.56)
80 years or above	–		–	–			–	–
Anxiety								
Meets clinical caseness	3.88**	(2.67, 5.64)	0.95	(0.56, 1.62)	12.65**	(6.64, 24.08)	2.49*	(1.01, 6.13)
Depression								
Meets clinical caseness	5.18**	(3.62, 7.40)	2.59**	(1.55, 4.33)	19.56**	(9.27, 41.28)	8.72**	(3.18, 23.91)
Loneliness								
Yes	4.70**	(3.23, 6.83)	2.54**	(1.63, 3.97)	3.90**	(2.06, 7.34)	0.81	(0.36, 1.84)
Mental health help-seeking								
Yes	2.92**	(2.05, 4.15)	1.46	(0.96, 2.23)	2.67*	(1.14, 4.92)	0.61	(0.29, 1.31)

The multivariate association between the correlates and group membership were then examined (see Table 3). The multinomial logistic regression model of diagnostic status was statistically significant (*χ*^*2*^ (64) = 296.91, *p* < 0.001). Positive correlates of moderate PGD included low income (two lowest income categories compared to highest), living in Northern Ireland (compared to living in England), living in a town (compared to rural), being aged 18–34 years (compared to ≥ 65 years), identifying as religious (compared to not religious), age of deceased being 0–17 years (compared to ≥ 80 years), less time since bereavement (compared to 10 + years since bereavement), depression (compared to not meeting caseness criteria), and loneliness (compared to not meeting caseness criteria). Correlates of strict PGD included religiosity (compared to not religious), younger age of the deceased (i.e., 0–17 years, 18–34 years, 35–59 years compared to ≥ 80 years), less time since the bereavement (i.e., 6–12 months, 1–2 years compared to ≥ 10 years), anxiety (compared to not meeting caseness criteria), and depression (compared to not meeting caseness criteria).

## Discussion

The current study had two primary aims: (1) report the levels of PGD symptoms, and estimate rates of moderate/strict PGD, and (2) identify sociodemographic, loss, and mental health related correlates of ICD-11 PGD (moderate and strict) using data from a large, nationally representative sample of UK adults.

### Levels of PGD symptoms and rates of ICD-11 PGD

Results demonstrated that common features of PGD in the UK population (at the moderate and strict level) include longing and preoccupation with the deceased, intense sadness, and loss of identity. A substantially smaller proportion of bereaved adults reported functional impairment associated with their PGD symptoms or persistence of PGD symptoms for an atypically long time relative to cultural norms, both of what are essential features of ICD-11 PGD. These findings are not surprising given that the symptoms comprising ICD-11 PGD are not considered inherently pathological but rather it is the persistence of symptoms and their ability to interfere with daily functioning which separates ICD-11 PGD from a “normal” grief response [27]. These findings are similar to prior studies where a relatively small albeit significant proportion of bereaved individuals meet criteria for probable diagnosis of PGD [e.g., 2, 5, 6, 28]. Hence, high endorsement of functional impairment and the cultural criterion would not be expected given that only a small proportion of bereaved individuals are postulated to experience an enduring and disabling grief response [2]. Findings suggested that the prevalence of ICD-11 PGD was between 2.4 and 8%. The prevalence of moderate PGD (i.e., 8%) fell within the lower range of strict PGD rates observed in prior studies [4, 5]. This may have been related to the small samples used in other studies and/or the sampling methods (which included the recruitment of participants from bereavement support groups [4, 5]). The proportion of participants who met probable caseness for PGD using the ‘strict approach’ was slightly higher than that observed in a general population sample of German adults [6], suggesting that different measures may produce divergent prevalence rates. That being said, the probable diagnostic rates observed in the present study align with a recent systematic review suggesting that 6.8 to 14.0% experience symptoms consistent with PGD [2]. However, the variability in prevalence rates across studies underscores the importance of determining where a threshold for ICD-11 PGD caseness should be set. In ICD-11, PGD belongs to the diagnostic category of stress-related disorders, alongside Posttraumatic Stress Disorder (PTSD), Complex PTSD (CPTSD), and Adjustment Disorder (AjD) [3] and each require an external stressor for diagnosis (a traumatic event in the case of PTSD and CPTSD, a psychosocial stressor or multiple stressors in the case of AjD, and the loss of a loved one in the case of PGD). The thresholds used in the self-report measures developed to assess these other stress-related disorders—the International Trauma Questionnaire [ITQ; 28] for ICD-11 PTSD and CPTSD and the International Adjustment Disorder Questionnaire [IADQ; 29] for ICD-11 AdJ—require that symptoms be present at least sometimes (i.e., a score of ≥ 3 on Likert scale of symptom frequency). These thresholds align to the ‘moderate’ algorithm for the IPGDS. Hence, it could be argued that the diagnostic algorithm employed to determine caseness of ICD-11 PGD should mirror these other stress-related disorders. Given that almost half (i.e., 40.6%) of the present sample reported their loss as occurring more than five years ago and that research has shown more recent losses to be associated with higher rates of PGD [e.g., 9] further research is required to understand the extent of PGD among recently bereaved adults.

### Sociodemographic, loss-related, and mental health correlates of PGD (moderate and strict)

Findings from the bivariate analyses highlighted several sociodemographic correlates of both moderate and strict PGD including low income, receiving government benefits, younger age of bereaved, and not being in a committed relationship. After adjusting for all other correlates, only low income and younger age of the bereaved remained significant correlates of moderate but not strict PGD. Specifically, participants in the two lowest income categories were over twice as likely to meet criteria for PGD than those in the highest income category. These effect sizes were much larger than those observed in prior studies assessing the association between low income and CMDs where ORs ranged from 1.18, for those earning between £100–£200 pounds per week (equivalent to our lowest income category), to 0.96 for those earning between £400–£500 pounds per week (equivalent to our second lowest income category) [30]. Moreover, the greater likelihood of moderate PGD among young adults aligns with the broader psychological literature evidencing a higher prevalence of mental disorders among young adults [31]. Contradicting the well-established gender gap in mental health [e.g., 32], no gender effects were observed in the current study. The absence of a gender effect may be considered in the context of a recent study investigating gender differences in trajectories of grief which found that although males and females were equally as likely to experience prolonged grief symptoms, males typically experienced acute PGD symptoms which abated with time, while females experienced worsening symptoms over time [33]. Hence, the commonly observed gender effects in mental health may not extend to PGD however, the disorder trajectory may differ in males and females. Other correlates of moderate PGD from the bivariate and multivariate analyses included living in NI as compared to England and living in a town as compared to rural areas. The former finding may be linked to “the Troubles” in NI, a thirty-year long period of political violence in NI which resulted in more than 3600 deaths [34], while the latter finding is consistent with literature indicating a lower prevalence of CMDs among rural inhabitants [35]. Overall, many of the sociodemographic correlates investigated were associated with increased risk of moderate but not strict PGD, suggesting that sociodemographic factors may be less predictive of more severe grief responses when considered in the context of other potentially more relevant factors such as loss-related and mental health factors.

Indeed, both bivariate and multivariate results demonstrated that loss-related correlates and psychological wellbeing were risk factors for moderate and strict PGD, with these effects being greatest for the latter group. Consistent with prior PGD research, bereavement recency was a risk factor for both moderate and strict PGD [9, 14]. Research has shown that time since trauma represents an important risk factor for PTSD and CPTSD [36], indicating that recency of the external stressor may represent a common risk factor among the stress-related disorders. Alternatively, data for this study was collected during the COVID-19 pandemic, and it is likely that some participants had experienced bereavements during the pandemic. There is a growing body of research illustrating a high prevalence of PGD among individuals bereaved due to COVID-19 [e.g., 11–13, 37], and hence further research is required to examine whether deaths that have occurred during the COVID-19 pandemic (both COVID-related and non-COVID related) have been associated with elevated prevalence of ICD-11 PGD. Consistent with prior research [e.g., 16, 17], younger age of the deceased was identified as a risk factor for moderate and strict PGD, and these effects were greatest for those reporting the death of a child. Similar to the death of a child, the death of a young or middle-aged adult is often unexpected and untimely which may lead to more maladaptive grief responses. It was interesting that religiosity (as compared to non-religiosity) was associated with increased risk of moderate and strict PGD, especially given that some prior research has shown those with strong spiritual beliefs demonstrate more adaptive mental health responses to grief [e.g., 38]. Further research is required to unpack the association between religiosity and ICD-11 PGD.

Finally, in terms of mental health correlates, depression, anxiety, loneliness, and mental health help-seeking were associated with both moderate and strict PGD in the unadjusted analyses. After controlling for other correlates, depression and loneliness remained significant correlates of moderate PGD while depression and anxiety remained significant correlates of strict PGD. Previous factor analytic work has shown that PGD, depression, and anxiety represent distinct yet highly correlated constructs [e.g., 39], and that the co-occurrence of these disorders is not uncommon. Similarly, there are several commonalities among the symptoms captured by the Loneliness Scale (i.e., lacking companionship, feeling left out, isolation from others) and the IPGDS (e.g., “*I feel that I lost a part of myself*”, “*I have difficulties engaging in activities*”, “*grief significantly interferes with my ability to work, socialize, or function in everyday life*”). Interestingly, anxiety uniquely predicted strict PGD while loneliness uniquely predicted moderate PGD in the multivariate analyses. This suggested that anxiety may be more prevalent among those with more severe grief symptoms while loneliness may be more relevant to those with moderate symptoms of grief. Future research may benefit from unpacking these associations, particularly given that understanding comorbidities associated with PGD remains an important research priority [40]. It is well-established that anxiety is a common response to bereavement due to the separation from a significant other, confrontation with one’s mortality, and exposure to stressors including financial adversity [41]. Indeed, research has shown how high levels of comorbidity between PGD and adult separation anxiety disorder (ASAD) [42] and generalized anxiety disorder [43]. It should be noted that intense loneliness is acknowledged as a PGD symptom in the text version of the DSM-5 [44] and as such, may be regarded a feature rather than a correlate of PGD. Whether intense loneliness should be listed as a symptom of PGD in the ICD-11 is a matter which warrants investigation.

### Strengths and limitations

Findings from this study should be considered in terms of several limitations. First, the cross-sectional nature of the data does not allow for conclusions to be drawn regarding causality. Second, the use of self-report measures are subject to several biases including misinterpretations of items and response categories [45]. Nevertheless, it is important to highlight that a clinician administered tool is not yet available. Third, although we aimed to include a wide range of sociodemographic, loss-related, and mental health correlates, it is likely that other potentially relevant indicators were excluded. Because the C19PRC assesses a broad range of social, economic, and mental health variables in the context of the COVID-19 pandemic, a thorough examination of grief-related variables was not possible. For instance, relationship to the deceased and cause of death has been shown to be important correlates of PGD [e.g., 14–16], which were not assessed within the C19PRC survey. Fourth, it should be noted that the current study did not investigate the psychometric properties of the IPGDS and hence, the validity of this measure within this particular sample is uncertain. Further research is now necessary to examine the psychometric properties of the IPGDS within this sample, especially given the paucity of such studies. Finally, only a small proportion of participants met probable caseness for 'strict PGD' and several categories of the correlates examined were relatively small (e.g., proportion of participants living in NI).This should be taken into account when evaluating the results as it is possible that it led to unstable estimates.

## Conclusion

In conclusion, this study is the first to examine the prevalence and correlates of ICD-11 PGD as measured via the IPGDS in a representative general-population sample of bereaved adults. This study highlights that approximately one in ten bereaved UK adults exhibit moderate levels of prolonged grief symptoms (i.e., moderate PGD) while one in forty exhibit high levels of prolonged grief symptoms (i.e., strict PGD). Considering the high prevalence of PGD in the general population, we recommend routine screening for PGD following a bereavement. Further exploration of the role of income, religiosity, area and country of residence, age of deceased, and mental health factors in predicting maladaptive grief responses is necessary. The variability in prevalence rates and risk factors when using the moderate and strict diagnostic algorithms emphasizes the need for an agreed upon expert consensus as to which algorithm to carry forward. This is especially imperative from a clinical perspective where prognosis and selection of clinical interventions are determined by diagnosis [46]. Future studies may benefit from determining the clinical meaningfulness of moderate PGD symptoms as compared to strict PGD symptoms to ensure that the algorithm employed to determine PGD caseness does not discount bereaved individuals in need of clinical intervention.

## Data Availability

The wave five data used in the current study is available at: https://osf.io/ducgs.
